# Comparative study between fluidless resuscitation with permissive hypotension using the impedance threshold device versus aggressive fluid resuscitation with Ringer lactate in a swine model of hemorrhagic shock

**DOI:** 10.1186/cc14254

**Published:** 2015-03-16

**Authors:** C Pantazopoulos, I Floros, N Archontoulis, D Xanthis, D Barouxis, N Iacovidou, T Xanthos

**Affiliations:** 1 Laiko General Hospital of Athens, Greece; 2 University of Athens, Medical School, Athens, Greece

## Introduction

Permissive hypotension, which results in avoidance of intravascular overpressure and thereby avoidance of platelet plug dislodgement early in the clotting mechanism, improves the results after trauma and hemorrhage. The research hypothesis is that augmentation of negative intrathoracic pressure with the use of an impedance threshold device (ITD) will improve hemodynamic parameters, without affecting permissive hypotension or causing hemodilution. On the other hand, aggressive resuscitation with Ringer lactate will cause hemodilution and intravascular pressures that are very high for permissive hypotension, capable of platelet plug dislodgement.

## Methods

Twenty anesthetized Landrace/Large-White pigs (19 ± 2 kg, 10 to 15 weeks) were subjected to a fixed hemorrhage (50% over 30 minutes). The pigs were randomly allocated into two groups (*n *= 10 per group). In group A, ITD was the only treatment for hypotension, while in group B, an intravenous administration of 1 | Ringer lactate was applied for treatment of hypotension. Hemodynamic parameters were continuously assessed for the first 30 minutes after blood loss.

## Results

Mean systolic arterial pressures (SAPs) 30 minutes after the intervention in each group were as follows: group A 80 ± 5 mmHg and group B 90 ± 4 mmHg. Maximum SAPs during the assessment period were: group A 89 ± 2 mmHg and group B 128 ± 5 mmHg. Mean pulse pressure was higher in the ITD group versus the fluid resuscitation group (*P *< 0.05). After the assessment period, mean hematocrit in group A was 24 ± 2%, while in group B it was 18 ± 1% (*P *< 0.001).

## Conclusion

In our study, the ITD increased SAP and pulse pressure without overcompensation. On the other hand, aggressive fluid resuscitation led to a significant increase of SAP >100 mmHg capable of clot dislodgement and in addition led to hemodilution.

